# Clinical Health Care during the COVID-19 Pandemic

**DOI:** 10.3390/jcm9113559

**Published:** 2020-11-05

**Authors:** Marco Luigetti, Giovanni Frisullo

**Affiliations:** 1 Fondazione Policlinico Universitario, “A. Gemelli” IRCCS, UOC Neurologia, Largo A Gemelli 8, 00168 Rome, Italy; giovanni.frisullo@policlinicogemelli.it; 2 Dipartimento di Neuroscienze, Università Cattolica del Sacro Cuore, 00168 Rome, Italy

**Keywords:** SARS-CoV2, COVID-19, healthcare system, neurological disorders, stroke, muscle, precision medicine

In December 2019, the current outbreak of the new Coronavirus 19 (CoV) was identified in Wuhan, China, and then quickly spread over the world. Europe has been involved since February 2020, Italy being the first country [1].

Patients with the new CoV have symptoms similar to those of severe acute respiratory syndrome CoV (SARSCoV) spread in 2002–2004: indeed, both viruses share the same receptor, angiotensin-converting enzyme 2 [2]. For this reason, the new virus was named SARS-CoV-2, and in February 2020, the World Health Organization (WHO) named it coronavirus disease 2019 (COVID-19) [3].

Infection in humans often leads to severe clinical manifestations and high mortality [3]. Several studies have described the clinical features: the disease often manifests with flu-like symptoms, and pulmonary complications are the main feature, but various clinical manifestations involving different organs or systems can be associated [3,4]. In general, extra-pulmonary signs or symptoms appear with the progression of the disease, but in some cases may represent the onset [4]. Viral tropism and host immune responses may explain these diverse manifestations, but the precise mechanisms have not been elucidated [5].

Numerous hematologic complications of SARS-CoV-2 infection have been reported: lymphopenia, coagulation disorders, increased venous thromboembolism, etc. [6].

Cardiac complications are common (~20–25%): in older patients with chronic comorbidities (e.g., arterial hypertension, diabetes, cardiac and cerebrovascular disorders), the prevalence and risk of death of severe COVID-19 are higher [7].

Gastrointestinal symptoms can be the onset of COVID-19, and they can precede respiratory manifestations of disease by 1–2 days [8].

Acute kidney injury is a frequent complication: direct infection by SARS-COV-2 with tubular injury, pigment casts due to rhabdomyolysis, endothelial damage, glomerular thrombi, cytokine storm and/or hemodynamic changes due to severe sepsis and multiorgan failure could be the causes [9].

The cutaneous manifestations are quite varied and occur frequently (>20%): morbilliform (maculopapular), urticarial, vesicular, pernio/chilblains-like and necrotic/livedoid are the most frequently detected lesions [10].

Finally, neurological manifestations of COVID-19 are frequent, being reported in about one third of patients: symptoms and signs may involve the nervous system at all levels, from the brain to muscle [11].

Considering the rampage through the body produced by the SARS-COV-2, the greatest COVID-19 impact was on the healthcare system, which has had to face new challenges due to unexpected and more severe clinical pictures with a multiorgan involvement. Moreover, the COVID-19 pandemic has had a different impact on the in-hospital and pre-hospital performance indicators of many care pathways. On the other hand, the COVID-19 pandemic and the resulting lockdown profoundly changed the lives of people everywhere and influenced attitudes and behaviors.

At present, COVID-19 has been declared a global pandemic, but our understanding of the disease is still limited. Given that COVID-19 patients can manifest with symptoms and signs involving the whole body, clinicians need to be involved, alert and prepared.

The aim of this Special Issue is to present clinical and scientific reports that improve our understanding of strategies capable of managing and addressing the COVID-19 pandemic. Authors can provide information about specific integrated care pathways or new treatment approaches to improve the management of SARS-Cov-2 infection.
